# Prevalence and factors associated with low functional mobility in older adults

**DOI:** 10.1002/agm2.12323

**Published:** 2024-06-28

**Authors:** Fernanda Nascimento de Oliveira, Eduarda Pereira Damião, Lucas dos Santos, Lucas Lima Galvão, Helen Rocha Machado, Rizia Rocha Silva, Sheilla Tribess, Jair Sindra Virtuoso Júnior, Douglas de Assis Teles Santos

**Affiliations:** ^1^ Department of Education, College of Physical Education State University of Bahia Teixeira de Freitas Brazil; ^2^ Graduate Program in Nursing and Health State University of Southwest Bahia Jequié Brazil; ^3^ Graduate Program in Physical Education Federal University of Triângulo Mineiro Uberaba Brazil; ^4^ Professional Master's Program in Collective Health State University of Bahia Salvador Brazil; ^5^ Faculty of Physical Education and Dance Federal University of Goiás Goiânia Brazil

**Keywords:** aging, epidemiology, health risk behaviors, public health

## Abstract

**Objective:**

To analyze the factors associated with low functional mobility in older adults residing in Alcobaça, BA.

**Methods:**

This is an epidemiological survey with a cross‐sectional design, conducted in 2015 with 473 older adults (62.4% women; mean age 70.2 ± 8.2 years) from Alcobaça, BA. The interview script addressed sociodemographic characteristics, health, and behavioral aspects. Functional mobility was assessed using the Short Physical Performance Battery (≤6 points). Inferential analyses were conducted using the Mann–Whitney U test and Poisson regression (with robust variance and estimation of prevalence ratios and their respective 95.0% confidence intervals).

**Results:**

The prevalence of low functional mobility was 9.6%, with associated factors including the use of alcoholic beverages (PR = 1.7, 95% CI: 1.01–1.13) and the number of repetitions in elbow flexion (PR = 1.01, 95% CI: 1.01–1.05). Additionally, older adults with low mobility had lower height, thigh circumference, and lower performance in handgrip strength tests, elbow flexion, and flexibility. They also spent more time in sedentary behavior and less time in physical activity compared to older adults with preserved mobility (*p* < 0.05).

**Conclusion:**

Older adults with low mobility exhibit poorer values in anthropometric parameters, lower performance in motor tests, spend less time engaged in physical activities, and more time in sedentary behavior.

## Graphical abstract

The study obtained a prevalence of low functional mobility was 9.6%, associated use of alcoholic beverages, number of repetitions in the elbow flexion, lower height, thigh circumference and lower performance in handgrip strength tests, elbow flexion, flexibility, more time in sedentary behavior, and less time in physical activity.

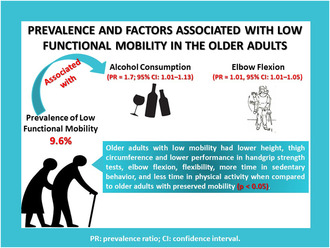

## 1 INTRODUCTION

The accelerated demographic transition, observed in both developed and developing countries, poses an important challenge to public health, as behavioral,^1^ psychological, and social changes occur throughout aging, which is experienced differently by each individual depending on the social, political, and economic context in which they are inserted.^2^ Therefore, there are several concerns about changes in the pattern of illness and their impacts on health conditions when considering longevity, such as functionality.

In this context, functional mobility can be conceptualized as the physical capacity that a mobile segment can reach within a range of motion to carry out a useful activity.^3^ Thus, its decline, which occurs with advancing age, results in decreased performance in carrying out daily activities.^4^

Limitations in the ability of older adults to carry out daily activities can be attributed to structural and functional deterioration, which affects most physiological systems. These include changes in muscle power, aerobic capacity, blood pressure, cardiovascular function, body composition, walking pattern, bone mineral density, balance, and mobility.^5^

Furthermore, low functional mobility can significantly impact health conditions, increasing the likelihood of morbidity, mortality, and hospitalization. This, in turn, leads to social and economic complications for older adults, their families, and the healthcare system.^6^

Therefore, considering that the Brazilian population is rapidly advancing toward an increasingly aging demographic profile, accompanied by various limitations that may have implications for older adults, the necessity of conducting epidemiological studies in different regions of Brazil emerges. This is due to the country's continental dimensions, where each location is influenced by various factors including lifestyle, dietary habits, and access to healthcare services.^7^

These factors can influence functional mobility, and investigating this outcome in each health territory can yield more specific information on the groups where this outcome is more prevalent. Consequently, it can provide valuable insights for health monitoring of older adults and facilitate the implementation of interventions such as balance, strength, and power training programs. These interventions have the potential to prevent and/or minimize the impact of functional decline and contribute to the care, promotion, and recovery of functionality and health conditions among the respective population. Thus, this study aimed to analyze the factors associated with low functional mobility in older adults residing in Alcobaça‐BA.

## 2 MATERIALS AND METHODS

### 2.1 Study design

This investigation is part of the “Longitudinal Health Study of the Elderly in Alcobaça”—ELSIA. This research is characterized as observational, analytical, with a cross‐sectional design, using exploratory methods (surveys and functional performance tests). Data were collected by academics and Physical Education professionals, duly trained, from July to September 2015.

### 2.2 Study site and participants

The study site was the municipality of Alcobaça, situated in the southern region of the State of Bahia, Brazil. According to the Social Indicators provided by the Brazilian Institute of Geography and Statistics,^8^ the estimated population in 2010 was 21,271 inhabitants, with 2047 individuals aged 60 or over.

The older adults (≥60 years) should not have a cognitive impairment, identified from the mini‐mental state examination (MMSE) (≤11 points), adapted for the Brazilian population,^9^ should not have severe difficulty in visual and auditory acuity, should not use wheelchairs, should not have severe sequelae of a cerebrovascular accident (CVA), with localized loss of strength, and should not have an end‐stage disease to participate in the study.

**FIGURE 1:**
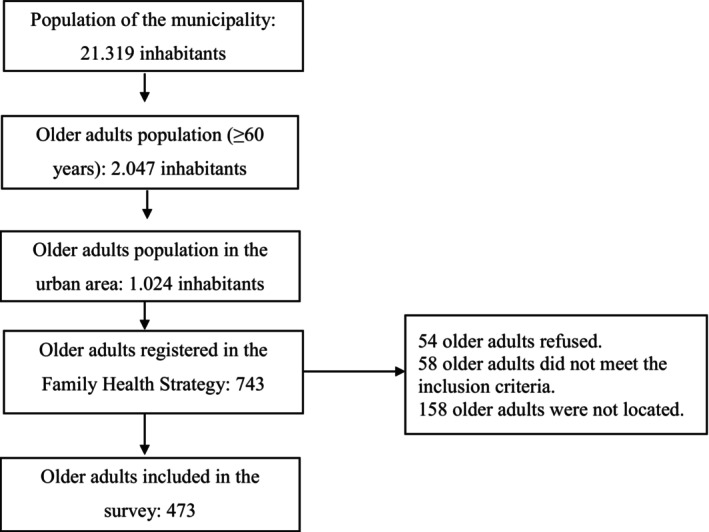
Diagram of decisions in the selection process of older adult participants in the study. Alcobaça‐BA, Brazil, 2015.

A census, conducted through the records of the Family Health Strategy, allowed identifying those 743 older adults who lived in the urban area of Alcobaça‐BA in 2015. However, according to the established criteria, the final sample consisted of 473 older adults (Figure 1).

The researchers used data provided by the Municipal Health Department of Alcobaça‐BA as a reference to conduct the health survey. Contact with the older adults was carried out through home visits. On this occasion, the objectives and procedures of the research were informed and the participation in the research was requested, which occurred voluntarily after signing the Free and Informed Consent Form.

The interview script was previously tested in a pilot study (to identify psychometric indices) and addressed sociodemographic characteristics and health and behavioral aspects.

### 2.3 Sociodemographic aspects

The sociodemographic variables addressed included years of study, with participants classified as having ≤4 years of study (no formal schooling) and >4 years of study (formal schooling); family arrangement, categorized as “with a partner” and “without a partner”; race/ethnicity, recorded in the categories: indigenous, brown, black, and white, based on self‐reporting; and occupation, divided as follows: paid work, pensioner, housewife, retired, and retired but still working.

### 2.4 Health aspects

Medication use was evaluated based solely on medications taken continuously and prescribed by a medical professional. These medications were classified into three categories: ≥3 medications, 1 to 2 medications, and no continuous medication use.^10^

Cognitive decline was assessed using the MMSE, an instrument designed to evaluate cognitive functioning and useful for monitoring changes over time. Older adults were dichotomized into categories of insufficient and sufficient cognitive function, with cutoff points of 19/20 for individuals without formal schooling and 23/24 for those with formal schooling.^9^

Depressive symptoms were screened using the short version of the Geriatric Depression Scale (GDS‐15), which has been translated and validated for the Brazilian population. The scale comprises 15 affirmative/negative questions related to various aspects such as satisfaction with life, happiness, and social interaction. The total score for GDS‐15 ranges from 0 to 15 points, with higher scores indicating a greater severity of depressive symptoms. A cut‐off point of six points or more was adopted to indicate the presence of depressive symptoms.^11^

Hospitalizations considered in the study encompassed the period of the last 12 months preceding data collection and were classified as follows: hospitalizations within the last 12 months, hospitalizations within the last 6 months, hospitalizations within the last 3 months, and no hospitalization. Additionally, older adults were asked about their medical history regarding cancer, arterial hypertension, and cerebrovascular accident, with response options being “yes” or “no.”

Systolic and diastolic blood pressure measurements were obtained at 5‐minute intervals using a digital sphygmomanometer (Omron—HEM 7113). The procedure was explained to the older adults, who were instructed to refrain from talking during the measurements. The cuff was positioned on the right arm at heart level, with the palm facing upward, the back and forearm supported, legs uncrossed, and feet flat on the floor. Two measurements were taken with an interval of 1 to 2 min, and additional measurements were performed only if the first two readings differed by more than 10 mmHg.^12^

### 2.5 Functional and behavioral aspects

Body mass was measured using a Wiso digital scale (model W721) with a capacity of 180 kg. Height was measured using a portable stadiometer with a precision of 0.1 cm. Arm circumference was measured on the posterior aspect of the arm, midway between the lateral projection of the acromial process and the inferior margin of the olecranon of the ulna. Thigh circumference was measured at the midpoint between the inguinal line and the upper edge of the patella. Both measurements were taken on the right side of the older adults using an anthropometric, flexible, and inelastic tape with a length of 2 m and a precision of 1 mm (Lange‐TBW, Cambridge, USA). Measurements were taken in triplicate, and the average value was considered for analysis.^13^

Muscle strength was measured using a SAEHAN hydraulic dynamometer (Saehan Corporation SH5001, Korea). The device was adjusted according to the gender of the participant, with setting 2 for women and setting 3 for men. The handgrip test was conducted with the participant using the arm they considered to be stronger. Participants stood upright with the arm extended away from the body and the elbow in extension. Two measurements were taken with a one‐minute interval between them, and the highest value obtained in kilogram‐force (kgf) was considered for analysis.^14^

Flexibility of the lower and upper limbs was assessed using the sit‐and‐reach test and the behind‐the‐back reach test from the Senior Fitness Test battery,^15^ utilizing a ruler with a precision of 0.1 cm and a total length of 30 cm as the measuring instrument. For the sit‐and‐reach test, the distance to the toes was recorded as zero, with distances anterior to the toes considered negative (−) and distances beyond the feet considered positive (+). For the behind‐the‐back reach test, the distance between the tips of the middle fingers was recorded as zero, with distances anterior to the fingers considered negative (−) and distances beyond the fingers considered positive (+), representing the measure of overlapping fingers/hand. Each test was performed three times, and the best performance was recorded.

The elbow flexion test was conducted using the dominant arm of the older adults, who were seated in a chair with a backrest and no armrests. Women used 2 kg dumbbells, while men used 4 kg dumbbells. Participants were instructed to perform as many repetitions as possible within a 30‐second period, which commenced at the evaluator's command. The evaluator provided a demonstration of how to perform the test prior to commencement.^15^

Measurement of habitual physical activity (PA) and time spent in sedentary behavior (SB) was conducted using the International Physical Activity Questionnaire (IPAQ), validated for the Brazilian older adult population.^16^, ^17^ PA was assessed using the first four IPAQ domains: leisure‐time physical activity, job‐related activity, transport‐related activity, and domestic activities.

SB was assessed by the amount of time spent sitting, identified through questions regarding the time spent sitting on a typical weekday and a typical weekend day. The total sitting time in minutes per day was determined by calculating the weighted average of sitting time on a weekday and a weekend day using the formula: [(time sitting on a weekday × 5 + time sitting on a weekend day × 2)/7].

Alcohol consumption was dichotomized as either yes or no. Smoking status was classified as follows: smokers (yes), individuals who quit smoking less than 12 months ago, individuals who quit smoking more than 12 months ago, and individuals who never smoked.

### 2.6 Assessment of the older adults’ mobility

The limitation of functional mobility was assessed using the Brazilian version of the short physical performance battery (SPPB). The adaptation of the SPPB to Brazilian culture resulted in a version that was easily comprehensible for both evaluators and older adults. This adaptation proved to be an instrument of easy and quick administration.^18^

The total SPPB score is calculated by summing the scores for each test (balance, gait speed, and sitting and standing up from a chair). Scores range from zero (indicating the worst performance) to 12 points (indicating the best performance).^18^ According to Guralnik et al.,^19^ SPPB scores from 0 to 3 points indicate disability or very poor performance, 4 to 6 points indicate low performance, 7 to 9 points indicate moderate performance, and 10 to 12 points indicate good performance. In this study, a dichotomous categorization was adopted, where the presence of functional limitation for lower limb mobility was determined by a score of ≤6 points.

The balance test comprises three different positions, with participants encouraged to maintain each position for a period of 10 s. Position A involves standing with feet together, position B involves standing with 1 foot placed farther in front of the other, and position C involves standing with 1 foot fully in front of the other.^19^

The walking test was employed to assess the performance of older adults in covering a distance of 2.44 m as quickly as possible. A wide tape was affixed to the floor to mark the designated distance, and a Kenko KK2008 Quartz Timer Digital stopwatch was utilized to record the time taken. Participants were given a prearranged verbal command (e.g., “attention, go”) to commence walking. Three consecutive measurements were taken, and the shortest time to cover the distance was recorded in seconds for analysis. Scores were assigned as follows: score 1 for a time greater than 6.52 s, score 2 for a time between 4.66 and 6.52 s, score 3 for a time between 3.62 and 4.65 s, and score 4 for a time less than 3.62 s.^20^

The sit‐to‐stand test was conducted to assess the strength and endurance of the lower limbs. A standard chair without armrests, featuring a rigid seat and a height of approximately 43 cm, was utilized. The chair was stabilized by leaning against a wall or otherwise secured for added safety. Participants began the test seated in the middle of the chair with their back straight, feet parallel to the ground, and forearms crossed over their chest. A prearranged verbal signal (e.g., “attention, go”) was given to prompt participants to stand up to an upright position and then return to the seated position, repeating this movement five times consecutively. The total time taken to complete five repetitions of standing up and sitting down was recorded.^20^

Participants scored 0 points if they were unable to complete five repetitions of standing up within 60 s. They received 1 point if they completed the test in 16.70 s or more, 2 points for a time between 13.70 and 16.69 s, 3 points for a time between 11.20 and 13.69 s, and 4 points for a time of 11.19 s or less.^20^

### 2.7 Statistical analysis

The Epidata software version 3.1b was utilized for constructing the database, while data analyses were performed using the statistical software SPSS 22.0 (Statistical Package for the Social Sciences). Descriptive statistical procedures, including absolute and relative frequencies, mean, median, standard deviation, and interquartile range, were employed for data analysis.

Initially, raw models were constructed using Poisson regression with robust variation, estimating prevalence ratios (PR) and their respective 95.0% confidence intervals (CI) to identify factors associated with low functional mobility. At this stage, variables with a significance level of at least 20.0% (*p* ≤ 0.20) were selected for inclusion in the multivariate analysis.

Subsequently, a multiple explanatory model was developed using the established strategy, where variables were introduced in blocks and controlled by age (in years), sex (male or female), and income (continuous variable). Sociodemographic aspects comprised block 1, health conditions were included in block 2, and anthropometric, hemodynamic, and functional parameters were incorporated in block 3.

Comparison of quantitative variables between groups with and without low mobility was performed using the Mann–Whitney U test, with non‐normal distributions identified using the Kolmogorov–Smirnov test. The significance level adopted for all analyses was 5.0% (*p* ≤ 0.05).

### 2.8 Ethical aspects

This study was conducted in accordance with the Declaration of Helsinki, in compliance with the determination of Resolution No. 466/2012 of the Brazilian National Health Council. It was also approved by the Human Research Ethics Committee of the Federal University of Triângulo Mineiro under Opinion No. 966.983/2015.

## 3 RESULTS

The study sample comprised 473 older adults, with 62.4% being women. The mean age of the participants was 70.2 ± 8.2 years, and the prevalence of low mobility was approximately 9.6%. Specifically, the mean age was 69.9 ± 8.1 years for women and 70.8 ± 8.3 years for men. Additionally, 68.4% of the participants had less than 4 years of schooling, 66.0% were hypertensive, and 47.8% reported alcohol consumption. Further characteristics of the participants are presented in Table 1.

**TABLE 1:** Descriptive analysis of the characteristics of the studied sample.

Variable	% of answers	*n*	%
Years of education	99.6		
>4 years		149	31.6
≤4 years		322	68.4
Family arrangement	99.8		
With a partner		217	46.0
Without a partner		255	54.0
Occupation	100.0		
Paid work		32	6.8
Pensioner		32	6.8
Housewife		21	4.4
Retired		311	65.8
Retired but still working		77	16.3
Use of medications	100.0		
≥3 medications		207	43.8
≤2 medications		167	35.3
None		99	20.9
Cognition	100.0		
Insufficient		236	49.9
Sufficient		237	51.1
Depressive symptoms	100.0		
Presence		56	11.8
Absence		417	88.2
Hospitalization	100.0		
Yes, in the last 12 months		33	0.7
Yes, in the last 6 months		24	5.1
Yes, in the last 3 months		23	4.9
No		393	83.1
Cancer	100.0		
Yes		07	1.5
No		466	98.5
Arterial hypertension	100.0		
Yes		312	66.0
No		161	34.0
CVA	100.0		
Yes		11	2.3
No		462	97.7
Consumption of alcoholic beverage	100.0		
Yes		226	47.8
No		247	52.2
Smoking	100.0		
Yes		56	11.8
Stopped less than 12 months ago		09	1.9
Stopped 12 months ago or more		175	37.0
Never		233	49.3

*Note*: Alcobaça‐BA, Brazil, 2015.

Abbreviations: CVA, cerebrovascular accident; *n*, number of participants; %, percentage.

Older adults with low mobility exhibited significantly lower height and thigh circumference compared to those without low mobility (Table 2). Additionally, the group with low mobility demonstrated poorer performance in motor tests, including handgrip strength and elbow flexion. They also spent more time in sedentary behavior and less time engaged in physical activity (*P* < 0.05).

**TABLE 2:** Comparison of anthropometric parameters and motor performance among older adults of both genders with and without low functional mobility.

Variable	Low mobility				
	Yes		No		*P*‐value
	Median	IQR	Median	IQR	
Body mass (kg)	64.00	20.6	67.20	19.9	0.110
Height (cm)	151.95	15.7	157.10	14.2	0.021
Arm circumference (cm)	28.65	6.7	29.20	5.3	0.056
Thigh circumference (cm)	46.05	9.4	48.30	8.0	0.019
FUL (cm)	−17.75	24.6	−10.00	13.4	0.001
FLL (cm)	−5.50	21.7	0.00	21.0	0.048
Handgrip strength (kgf)	16.00	11.0	23.00	12.0	<0.001
Elbow flexion (reps)	11.00	7.0	19.00	7.0	<0.001
Total PA (minutes/week)	0.00	82.5	210.00	450.0	<0.001
Total SB (minutes/day)	590.00	250.1	404.14	190.7	<0.001
Systolic blood pressure (mmHg)	152.50	33.0	143.00	26.0	0.008
Diastolic blood pressure (mmHg)	77.50	20.0	79.00	15.0	0.241

*Note*: Alcobaça‐BA, Brazil, 2015.

Abbreviations: cm, centimeters; FLL, flexibility of the lower limbs; FUL, flexibility of the upper limbs; IQR, interquartile range; kg, kilogram; kgf, kilogram‐force; mmHg, millimeters of mercury; PA, physical activity; SB, sedentary behavior.

Table 3 indicates that all studied variables had a significance level of ≤20.0% in the raw analysis, thus warranting their inclusion in the multivariate analysis. The multivariate analysis revealed that the use of alcoholic beverages and the number of repetitions in elbow flexion were associated with a 7.0% (95% CI: 1.016–1.132) and 1.0% (95% CI: 1.016–1.018) higher probability, respectively, of the studied outcome.

**TABLE 3:** Raw and adjusted prevalence ratio (PR) for the independent variables relative to low mobility in older adults.

Variable	Low mobility			
	Raw analysis		Multivariate analysis	
	PR (95% CI)	*P*‐value	PR (95% CI)	*P*‐value
*Block 1—Sociodemographic conditions*				
Years of education		0.008		0.694
>4 year	1		1.01 [0.93–1.10]	
≤4 year	2.15 [1.01–3.0]		1	
Family arrangement		0.032		0.244
With a partner	1.03 [1.01‐1.06]		0.96 [0.90–1.02]	
Without a partner	1		1	
Occupation		<0.001		0.261
Paid work	0.99 [0.96–1.02]		0.91 [0.83–0.99]	
Pensioner	0.92 [0.85–0.99]		0.96 [0.83–1.09]	
Housewife	0.98 [0.93–1.03]		0.98 [0.88–1.09]	
Retired	0.95 [0.92–0.97]		0.99 [0.94–1.05]	
Retired but still working	1		1	
*Block 2—Health conditions*				
Use of medications		0.009		0.889
≥3 medications	0.95 [0.92–0.98]		0.98 [0.90–1.07]	
≤2 medications	0.98 [0.9–1.01]		0.98 [0.90–1.06]	
None	1		1	
Cognition		0.189		0.108
Insufficient	0.98 [0.95–1.01]		1.04 [0.98–1.11]	
Sufficient	1		1	
Depressive symptoms		0.031		0.140
Presence	0.93 [0.87–0.99]		0.90 [0.79–1.03]	
Absence	1		1	
Hospitalization		0.149		0.884
Yes, in the last 12 months	0.92 [0.84–1.00]		0.94 [0.79–1.10]	
Yes, in the last 6 months	0.97 [0.90–1.05]		0.97 [0.84–1.13]	
Yes, in the last 3 months	0.95 [0.86–1.03]		0.97 [0.82–1.15]	
No	1		1	
Cancer		<0.001		0.056
Yes	1.05 [1.0–1.07]		1.05 [0.97–1.13]	
No	1		1	
Arterial hypertension		0.151		0.158
Yes	0.98 [0.95–1.01]		0.84 [0.60–1.18]	
No	1		1	
CVA		0.177		0.321
Yes	0.89 [0.75–1.05]		0.84 [0.60–1.18]	
No	1		1	
Systolic blood pressure	0.99 [0.99–1.00]	0.009	0.99 [0.99–1.00]	0.209
*Block 3—Functional and behavioral aspects*				
Body mass (kg)	1.01 [1.00–1.02]	0.006	0.99 [0.99–1.00]	0.696
Height (cm)	1.01 [1.00–1.03]	0.047	0.99 [0.99–1.00]	0.662
Arm circumference (cm)	1.04 [1.00–1.07]	0.036	0.99 [0.99–1.00]	0.374
Thigh circumference (cm)	1.02 [1.00–1.04]	0.012	0.99 [0.98–1.00]	0.090
FUL (cm)	1.03 [1.01–1.05]	0.001	1.00 [1.00–1.00]	0.293
FLL (cm)	1.01 [1.00–1.02]	0.019	1.00 [0.99–1.00]	0.683
Handgrip strength (kgf)	1.04 [1.02–1.06]	<0.001	1.00 [0.99–1.00]	0.301
Elbow flexion (rep)	1.01 [1.00–1.01]	<0.001	1.01 [1.01–1.05]	<0.001
Total PA (min/wk)	1.00 [1.00–1.00]	<0.001	1.00 [1.00–1.00]	0.123
Total SB (min/day)	1.00 [1.00–1.00]	<0.001	1.00 [0.99–1.00]	0.002
Consumption of alcoholic beverage		<0.001		0.011
Yes	1.06 [1.02–1.09]		1.07 [1.01–1.13]	
No	1		1	
Smoking		0.025		0.375
Yes	1.04 [1.00–1.08]		1.00 [0.91–1.09]	
Stopped less than 12 months ago	0.93 [0.79–1.11]		0.81 [0.57–1.16]	
Stopped 12 months ago or more	1.04 [1.01–1.07]		1.03 [0.98–1.09]	
Never	1		1	

*Note*: Alcobaça‐BA, Brazil, 2015.

Abbreviations: CI, confidence interval; cm, centimeters; CVA, cerebrovascular accident; FLL, flexibility of the lower limbs; FUL, flexibility of the upper limbs; kg, kilogram; kgf, kilogram‐force; min/day, minutes per day; min/wk, minutes per week; PA, physical activity; PR, prevalence ratio; rep, repetition; SB, sedentary behavior.

## 4 DISCUSSION

The group with low mobility exhibited lower values for height and thigh circumference. This observation may be attributed to factors such as a reduction in the plantar arch and flattening of the vertebral discs, leading to a decrease in an individual's height over the years. Furthermore, older adults with low mobility tended to be older and thus exhibited a greater severity of age‐related changes.^5^

The measurement of thigh circumference serves as an indicator of muscle mass. Therefore, older adults with low mobility exhibited a smaller muscle mass, as aging contributes to a decline in the performance of the neuromuscular system, consequently increasing the risk of functional limitation.^5^, ^14^, ^21^

Physical performance tests revealed lower flexibility in both upper and lower limbs among older adults with low functional mobility. Flexibility, a crucial component of health‐related physical fitness, is essential for activities of daily living. Prolonged inactivity can lead to alterations in various organs and systems. In this scenario, reduced functional mobility may have contributed to muscle shortening, resulting in decreased flexibility. Furthermore, advancing age is a factor that can impact an individual's flexibility, as tendon stiffness tends to increase with age, consequently limiting joint mobility.^22^

Older adults with low mobility also exhibited weaker handgrip strength compared to those with preserved mobility. Similarly, Moreira et al.^23^ conducted a population‐based study in Alfenas‐MG, demonstrating that handgrip strength was inversely associated with functional dependence in older adults (OR: 0.20; 95% CI: 0.02–0.06).

Soares et al.^24^ conducted an investigation involving elbow flexion with 26 older adults using the timed up and go (TUG) test to assess functional mobility. The results revealed a negative correlation between the number of repetitions in elbow flexion and the functional mobility of older adults (*r* = −0.58, *p* < 0.001), indicating that higher muscle strength was associated with better performance in the TUG execution. Conversely, the study also found that repetitions in elbow flexion were positively associated with low mobility. This outcome is likely explained by a reduction in muscle strength that occurs with advancing age, resulting in decreased mobility.^5^

Regarding total physical activity time, Gomes,^25^ employed a direct measurement method (accelerometers) in a cross‐sectional study involving 543 older adults. The study found that 60.39% of those evaluated with impaired mobility had a low level of physical activity, whereas this number was only 20.26% among older adults without impaired mobility (*p* < 0.0001). Furthermore, this association remained significant after adjustments in the multiple hierarchical models (OR: 3.49; 95% CI: 2.00–6.13).

A longitudinal study conducted with 1635 Americans at risk for functional disability found that the group of older adults who received physical activity (PA) intervention with moderate intensity significantly reduced limitations in mobility compared to the group that received only lectures over a period of 2.6 years (HR: 0.82; 95% CI: 0.69–0.98).^26^

However, in the present investigation, older adults with low mobility demonstrated significantly longer exposure to sedentary behavior. This finding suggests that high sedentary time may be one of the contributing variables to a decline in functionality. As individuals age, there is an inevitable decline in biological functions, and this decline is often associated with prolonged sedentary behavior, which can increase the risk factors for hypokinetic diseases in the long or medium term. Therefore, the literature suggests that reducing sitting time, for example, can mitigate health issues and contribute to improved functional mobility.^27^

In this study, systolic blood pressure was higher among older adults with low mobility. Similarly, observational epidemiological research conducted in Cocal‐PI identified hypertension as one of the variables associated with low performance in the TUG test.^28^

Regarding the consumption of alcoholic beverages, a study with 3470 older adults conducted in Taiwan showed that frequent alcohol consumption was identified as harmful to mobility function in older adults.^29^ Conversely, in Spain, a study using two cohorts of older adults showed that moderate alcohol consumption was a protective factor against limiting mobility (OR: 0.80; 95% CI: 0.63 and 0.97).^30^

Although this consumption is harmful to people's health, in some cases, it may be associated with higher performance in social activities and better functional conditions.^30^ However, this substance can negatively interfere with functional, cognitive, and psychomotor capacity, increasing older adults' exposure to accidents, injuries, and institutionalization.^29^

This study has limitations, such as acquiring some information through self‐report, which may be subject to memory bias even after excluding older adults with low performance in MMSE. Furthermore, the use of the IPAQ, a subjective measure of behavioral assessment, may not be the most reliable, presenting limitations to the study. However, its strength lies in the measurement of muscle strength through handgrip, considered the gold standard method, as well as the evaluation of the outcome through a battery of tests addressing different aspects related to functional performance.

## 5 CONCLUSION

The evidence obtained indicates that older adults with low mobility exhibited poorer anthropometric parameter values, lower performance in motor tests, less time engaged in physical activity, and higher exposure to sedentary behavior. Additionally, low mobility was positively associated with alcohol consumption and the number of repetitions in elbow flexion.

## 6 RELEVANCE FOR CLINICAL PRACTICE

A pertinent clinical practice suggested by the results of the text involves implementing interventions focused on enhancing mobility in older adults. Based on the identified associations, such as low mobility being positively correlated with alcohol consumption and the number of repetitions in elbow flexion, clinical strategies can be devised to enhance mobility in this demographic.

For instance, an intervention program could be devised to encourage older adults to decrease alcohol intake by offering information about associated risks and the advantages of reducing or abstaining from alcohol. Furthermore, tailored strength training protocols for elbow flexion, incorporating exercises for muscle strengthening and flexibility, along with guidance on increasing overall physical activity and reducing sedentary behavior, can be introduced to enhance upper limb functional capacity.

This pertinent clinical practice aims to address the specific factors linked to low mobility in older adults by providing targeted interventions to improve mobility‐related outcomes and foster healthy aging.

## AUTHOR CONTRIBUTIONS

ST, JSVJ, and DATS made substantial contributions to the conception and design of the study. LLG, RRS, ST, JSVJ, and DATS collected the data. LLG and DATS performed the statistical analysis and interpretation of the data. FNO, EPD, LS, HRM, JSVJ, and DATS made substantial contributions to the writing of the manuscript. All authors critically revised the intellectual content of the manuscript and approved the final version as submitted.

## FUNDING INFORMATION

This study was financed in part by the Coordenação de Aperfeiçoamento de Pessoal de Nível Superior—Brasil (CAPES), with graduate scholarship and supported by Conselho Nacional de Desenvolvimento Científico e Tecnológico (MCTI/CNPQ/Universal 14/2014, grant number: 448184/2014–1).

## CONFLICT OF INTEREST STATEMENT

The authors declare that there is no conflict of interest.

## LOCAL DEVELOPMENT

Universidade do Estado da Bahia (UNEB), Teixeira de Freitas, Bahia, Brazil.

## References

[1] United Nations Department of Economic and Social Affairs . (2021). Global Population Growth and Sustainable Development. UN DESA/POP/2021/TR/NO.2.

[2] Dziechciaż M , Filip R . Biological psychological and social determinants of old age: bio‐psycho‐social aspects of human aging. Ann Agri Environ Med. 2014;21(4):835‐838. doi:10.5604/12321966.1129943 25528930

[3] Lenardt MH , Carneiro NHK , Binotto MA , Willig MH , Lourenço TM , Albino J . Fragilidade e qualidade de vida de idosos usuários da atenção básica de saúde. Rev Bras Enferm. 2016;69(3):478‐483. doi:10.1590/0034-7167.2016690309I 27355296

[4] Grimmer M , Riener R , Walsh CJ , Seyfarth A . Mobility related physical and functional losses due to aging and disease—a motivation for lower limb exoskeletons. J NeuroEng Rehab. 2019;16(1):1‐21. doi:10.1186/S12984-018-0458-8 PMC6318939 30606194

[5] Tieland M , Trouwborst I , Clark BC . Skeletal muscle performance and ageing. J Cachexia Sarcopenia Muscle. 2018;9(1):3‐19. doi:10.1002/jcsm.12238 29151281 PMC5803609

[6] Torres‐de Araújo JR , Tomaz‐de Lima RR , Ferreira‐Bendassolli IM , Costa‐de Lima K . Functional, nutritional and social factors associated with mobility limitations in the elderly: a systematic review. Salud Publica Mex. 2018;60(5):579‐585. doi:10.21149/9075 30550119

[7] Rodrigues Barbosa AI , Marchesan MI , Vieira Guimarães AI , et al. Anthropometric indicators and their adequacy in older adults from two towns in distinct Brazilian regions. MedicalExpress. 2015;2(6):M150605. doi:10.5935/MEDICALEXPRESS.2015.06.05

[8] IBGE . Censo Demográfico 2010: Sinopse. Instituto Brasileiro de Geografia e Estatística; 2010.

[9] Almeida OP . Mini exame dos estado mental e o diagnóstico de demência no Brasil. Arq Neuropsiquiatr. 1998;56(3B):605‐612. doi:10.1590/S0004-282X1998000400014 9850757

[10] Maher RL , Hanlon J , Hajjar ER . Clinical consequences of polypharmacy in elderly. Expert Opin Drug Saf. 2014;13(1):57‐65. doi:10.1517/14740338.2013.827660 24073682 PMC3864987

[11] Almeida OP , Almeida SA . Confiabilidade da versão Brasileira da escala de depressão em geriatria (GDS) versão reduzida. Arq Neuropsiquiatr. 1999;57(2 B):421‐426. doi:10.1590/s0004-282x1999000300013 10450349

[12] Barroso WKS , Rodrigues CIS , Bortolotto LA , et al. Brazilian guidelines of hypertension—2020. Arq Bras Cardiol. 2021;116(3):516‐658. doi:10.36660/ABC.20201238 33909761 PMC9949730

[13] Lohman TJ , Roache AF , Martorell R . Anthropometric standardization reference manual. J Am Diet Assoc. 1988;24:952. doi:10.1249/00005768-199208000-00020

[14] dos Santos L , dos Santos Santana P , da Silva Caires S , et al. Força e massa muscular em idosos do Nordeste brasileiro. Res Soc Develop. 2021;10(14):1‐13. doi:10.33448/rsd-v10i14.22270

[15] Rikli RE , Jones CJ . Development and validation of a functional fitness test for community‐residing older adults. J Aging Phys Act. 1999;7(2):129‐161. doi:10.1123/JAPA.7.2.129

[16] Benedetti TRB , Antunes PDC , Rodriguez‐Añez CR , Mazo GZ , Petroski ÉL . Reprodutibilidade e validade do Questionário Internacional de Atividade Física (IPAQ) em homens idosos. Rev Bras Med Esporte. 2007;13(1):11‐16. doi:10.1590/s1517-86922007000100004

[17] Benedetti TRB , Mazo GZ , de Barros MVG . Aplicação do Questionário Internacional de Atividades Físicas para avaliação do nível de atividades físicas de mulheres idosas: validade concorrente e reprodutibilidade teste‐reteste. Rev Bras Ciênc Mov. 2004;12(1):25‐34. doi:10.18511/rbcm.v12i1.538

[18] Nakano MM . Brazilian Version of the Short Physical Performance Battery—SPPB: Cross‐Cultural Adaptation and Reliability Study. Thesis; 2007. [Unversidade estadual de Campinas]. https://www.repositorio.unicamp.br/acervo/detalhe/396756

[19] Guralnik JM , Ferrucci L , Simonsick EM , Salive ME , Wallace RB . Lower‐extremity function in persons over the age of 70 years as a predictor of subsequent disability. N Engl J Med. 1995;332(9):556‐562. doi:10.1056/NEJM199503023320902 7838189 PMC9828188

[20] Cruz‐Jentoft AJ , Bahat G , Bauer J , et al. Sarcopenia: revised European consensus on definition and diagnosis. Age Ageing. 2019;48(1):16‐31. doi:10.1093/AGEING/AFY169 30312372 PMC6322506

[21] dos Santos L , Miranda CGM , de Souza TCB , Brito TA , Fernandes MH , Carneiro JAO . Body composition of women with and without dynapenia defined by different cut‐off points. Rev Nutr. 2021;34:e200084. doi:10.1590/1678-9865202134E200084

[22] Correia M , Menêses A , Lima A , Cavalcante B , Ritti‐Dias R . Efeito do treinamento de força na flexibilidade: uma revisão sistemática. Rev Bras Ativ Fis Saúde. 2014;19(1):3. doi:10.12820/RBAFS.V.19N1P3

[23] Moreira LB , da Silva SLA , de Castro AEF , et al. Fatores associados a capacidade funcional de idosos adscritos à Estratégia de Saúde da Família. Ciênc Saúde Colet. 2020;25(6):2041‐2050. doi:10.1590/1413-81232020256.26092018 32520252

[24] Soares AV , Marcelino E , Maia KC , Gomes N , Junior B . Relation between functional mobility and dynapenia in institutionalized frail elderly. Einstein. 2017;15(3):278‐282. doi:10.1590/S1679-45082017AO3932 29091148 PMC5823040

[25] Gomes IC . Prevalência do baixo nível de atividade física e sua associação no comprometimento da mobilidade e risco para óbito em idosos residentes no município de São Paulo. 2016 [Universidade de São Paulo]. http://www.teses.usp.br/teses/disponiveis/6/6132/tde‐12052016‐150330/publico/IgorConteratoGomesREVISADA.pdf

[26] Pahor M , Guralnik JM , Ambrosius WT , et al. Effect of structured physical activity on prevention of major mobility disability in older adults: the LIFE study randomized clinical trial. JAMA. 2014;311(23):2387‐2396. doi:10.1001/JAMA.2014.5616 24866862 PMC4266388

[27] Hamer M , Stamatakis E . Screen‐based sedentary behavior, physical activity, and muscle strength in the English longitudinal study of ageing. PLoS ONE. 2013;8(6):e66222. doi:10.1371/JOURNAL.PONE.0066222 23755302 PMC3670922

[28] Nascimento Da Silva L , Dayanne M , Ribeiro A , Oliveira SB , Carlos J , Silva A . Influência dos requisitos cinéticos funcionais e desfechos de saúde na mobilidade funcional de idosos. Rev Pesqui Fisioter. 2018;8(4):489‐496. doi:10.17267/2238-2704rpf.v8i4.2126

[29] Lêng CH , Der Wang J . Long term determinants of functional decline of mobility: an 11‐year follow‐up of 5464 adults of late middle aged and elderly. Arch Gerontol Geriatr. 2013;57(2):215‐220. doi:10.1016/J.ARCHGER.2013.03.013 23608344

[30] León‐Muñoz LM , Guallar‐Castillón P , García‐Esquinas E , Galán I , Rodríguez‐Artalejo F . Alcohol drinking patterns and risk of functional limitations in two cohorts of older adults. Clin Nutr (Edinburgh, Scotland). 2017;36(3):831‐838. doi:10.1016/J.CLNU.2016.05.005 27256558

